# Can Spleen T1 Mapping Be Used as a Marker of Adenosine Response in Cardiac MRI?

**DOI:** 10.1002/nbm.70430

**Published:** 2026-10-29

**Authors:** Sergio M. Solis‐Barquero, Ana Ezponda, Verónica Aramendía‐Vidaurreta, Marta Vidorreta, Marina Pascual, Gorka Bastarrika, Maria A. Fernández‐Seara

**Affiliations:** ^1^ Department of Radiology Clinica Universidad de Navarra Pamplona Spain; ^2^ IdiSNA, Instituto de Investigación Sanitaria de Navarra Pamplona Spain; ^3^ Department of Diagnostic and Therapeutic Imaging, School of Health Technologies University of Costa Rica; ^4^ Siemens Healthcare Madrid Spain; ^5^ Department of Cardiology Clinica Universidad de Navarra Pamplona Spain

**Keywords:** adenosine, multiparametric magnetic resonance imaging, myocardial perfusion imaging, perfusion magnetic resonance imaging, spleen

## Abstract

Splenic switch‐off, defined as the visible reduction in splenic perfusion during adenosine stress, can be used as a marker of adequate adenosine response in cardiac stress MRI studies, which can be assessed after the study. Moreover, there is some evidence regarding the use of T1 mapping for the evaluation of splenic switch‐off during the study. The aim of this work is to further investigate splenic T1 mapping as a method for quantitatively assessing splenic switch‐off, given the limited existing data. Patients with suspected coronary artery disease were scanned at 1.5 T with the routine clinical protocol for stress perfusion cardiac magnetic resonance (CMR), including a T1 mapping sequence at rest and during adenosine stress. Visual assessment of first‐pass images was used as the reference standard for detecting splenic switch‐off. Semi‐quantitative analysis of splenic perfusion was performed based on the signal intensity curves and signal changes (ΔSI) were calculated. T1 maps were used to obtain T1 value changes in the spleen from rest to stress (ΔT1_spleen_). Statistical analysis was assessed using an align rank transform followed by a mixed analysis of variance, with Bonferroni corrected post hoc tests. A total of 74.5% (*n* = 35/47) of analyzed patients experienced splenic switch‐off according to the visual assessment. Statistically significant differences were found in splenic T1 between rest and stress only in the switch‐off group (*p* < 0.001), with lower T1 values during stress. There was significant positive weak correlation (ρ_s_ = 0.37, *p* = 0.01) between splenic ΔT1 and ΔSI based on first‐pass. ROC curves demonstrated that ΔT1_spleen_ classified the failed switch‐off and positive switch‐off groups with a specificity of 75% and a sensitivity of 80% (AUC: 81.2%, 95% CI: 66.7%–95.8%). This work indicates that the splenic T1 value change has the potential to identify splenic switch‐off during adenosine stress CMR in a clinical population.

## Graphical abstract

Patients with suspected coronary artery disease were scanned at 1.5 T with the routine clinical protocol for CMR. T1 mapping sequence at rest and during stress was included in which the spleen was visible. Splenic switch‐off was visually assessed in first‐pass images. Splenic T1 value change has the potential to identify splenic switch‐off during adenosine stress CMR in a clinical population.

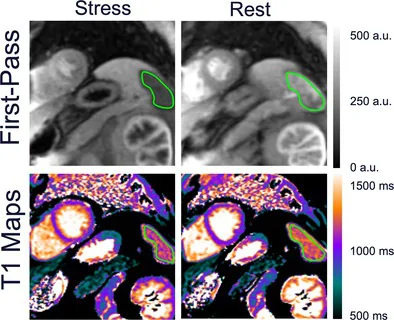

## 1 Introduction

In patients with coronary artery disease (CAD), stress perfusion cardiac magnetic resonance (CMR) is commonly used for the detection of myocardial ischemia. Ischemia detection is crucial as it guides clinical decision‐making since the targeted revascularization of significant coronary stenosis may improve clinical outcomes.

In perfusion CMR, a pharmacological vasodilator is used to increase myocardial blood flow (MBF). Typically, pharmacological stress agents such as adenosine are recommended [1], with adenosine being the most used and widely acceptable [2], but it has some concerns.

Adenosine has been related to false‐negative perfusion CMR results, with a false‐negative rate between 0% and 16% (depending on the report) [3] and at least one‐third of those can be associated with an inadequate adenosine response [4].

To control the adenosine response, CMR guidelines [1] recommend observing an increase in heart rate of at least 10 beats‐per‐minute after 3 minutes of infusion, or a reduction in the systolic blood pressure > 10 mmHg. However, these biomarkers may be poor surrogates of the hyperemic effect in the myocardium since these physiological responses do not necessarily reflect an increase in myocardial perfusion [5].

Splenic switch‐off, first described by Manisty et al. as the visible attenuation in splenic perfusion during adenosine stress, may be an alternative marker of adequate adenosine response [6]. This effect is attributed to adenosine A_1_ receptor‐mediated contraction of the splenic smooth muscle [7], which results in a decrease of blood flow and volume to the splenic tissue. Although it was first observed using contrast first‐pass imaging [6, 8, 9], this approach does not allow corrective action during the imaging study, as additional gadolinium administration to repeat the stress acquisition is not feasible. However, the effect has been demonstrated using other imaging techniques such as ASL [10], PET [11], and through a reduction in the T1 longitudinal relaxation time of the spleen from rest to stress [12].

The reduction in T1 during splenic switch‐off is likely related to the decrease in blood flow and associated reduction in blood volume. Because T1 relaxation times are sensitive to tissue water content and its compartmentalization [13], a reduction in the vascular water fraction within the spleen could lead to lower measured T1 values [14].

In routine CMR exams, T1 maps are commonly acquired in a short‐axis subset of slices of the left ventricle, in which the spleen is typically visible. Mean T1 values of the spleen can be obtained from these T1 maps acquired during rest and stress and may depict the splenic switch‐off through a significant reduction in T1 values during stress and can be measured during the acquisition.

In a previous single‐center study, a reduction in T1 values indicated splenic switch‐off with high sensitivity (90%), specificity (88%), and accuracy (90%) [12]. However, the same authors later claimed that this result could not be independently reproduced [15].

In another study, a drop in T1 was able to detect splenic switch‐off with lower specificity (63%) and accuracy (83%) [16]. To the best of our knowledge, only these two studies to date have evaluated the performance of splenic T1 mapping as a switch‐off marker. The aim of this study was to investigate T1 mapping as a quantitative method for assessing splenic switch‐off in a cohort of patients undergoing adenosine stress perfusion CMR.

## 2 Materials and Methods

### 2.1 Participants and Study Design

The study protocol was approved by the Research Ethics Committee of the Clinica Universidad de Navarra. All participants gave written informed consent prior to the study and voluntarily accepted to participate.

Participants were prospectively recruited between May 2023 and February 2024. General inclusion criteria were patients older than 18 years, with suspected or known CAD, who were scheduled for a stress perfusion CMR examination. Patients were scheduled for stress perfusion CMR examination based on institutional clinical criteria, including intermediate to high pre‐test probability of CAD, inability to perform or inconclusive prior stress testing, or known CAD with suspected recurrent ischemia. The general exclusion criteria were usual contraindications for CMR, such as pacemakers and hemodynamic instability, characterized by fluctuations in blood pressure, heart rate, or cardiac output, and impaired renal function, which could make the test unsafe for the patient.

### 2.2 MRI Acquisition

Patients were scanned in a 1.5 T Aera MRI scanner (Siemens Healthineers, Forchheim, Germany) with an 18‐channel body and 32‐channel spine coils. The routine clinical protocol for the stress perfusion CMR examination consisted of cardiac localizers followed by cine acquisitions in the long axis in four‐, three‐, and two‐chamber planes; native T1 and T2 mapping in the subset of short axis slices (i.e., basal, mid, and apical) and a long‐axis four‐chamber view; balanced steady‐state free precession (bSSFP) contrast first‐pass perfusion imaging during stress after adenosine infusion; post‐stress cine in the complete set of short axis slices; bSSFP contrast first‐pass perfusion imaging at rest, 10 min after stress acquisition; late gadolinium enhancement (LGE); and post‐contrast T1 mapping.

For this study, a single‐slice (mid‐ventricular, short‐axis) T1 mapping sequence during stress was added to the routine clinical protocol, before the acquisition of perfusion first‐pass images, to avoid measurements being affected by T1 reduction due to the gadolinium‐based contrast agent (GBCA).

A fixed adenosine (Adenoscan 30 mg/10 mL, Sanofi, Barcelona) dose of 140 μg/kg/min was injected intravenously under the direct supervision of a cardiologist. Heart rate and blood pressure were monitored during the whole study for patient safety. Patients were asked to self‐report possible side effects of the adenosine.

T1 images during stress were acquired approximately 3 min after the start of adenosine infusion, in that order. The acquisition of these images lasted less than 1 min. The first‐pass stress perfusion sequence was acquired immediately at the end of stress T1 mapping, after verifying the patient positive increase in heart rate (≥ 10 bpm). Time for image acquisition is an approximation since it varied due to heart rate differences. Heart rate was annotated from the ECG tracing displayed on the MRI console before the adenosine infusion (i.e., at rest) and at the beginning of the first‐pass acquisition in stress. Blood pressure and patient‐reported symptoms data were not recorded for research purposes but were controlled and attended during the scan. Adenosine infusion was stopped right after the last first‐pass images were acquired. The complete adapted protocol used in the study is graphically shown in Figure 1.

**FIGURE 1:**
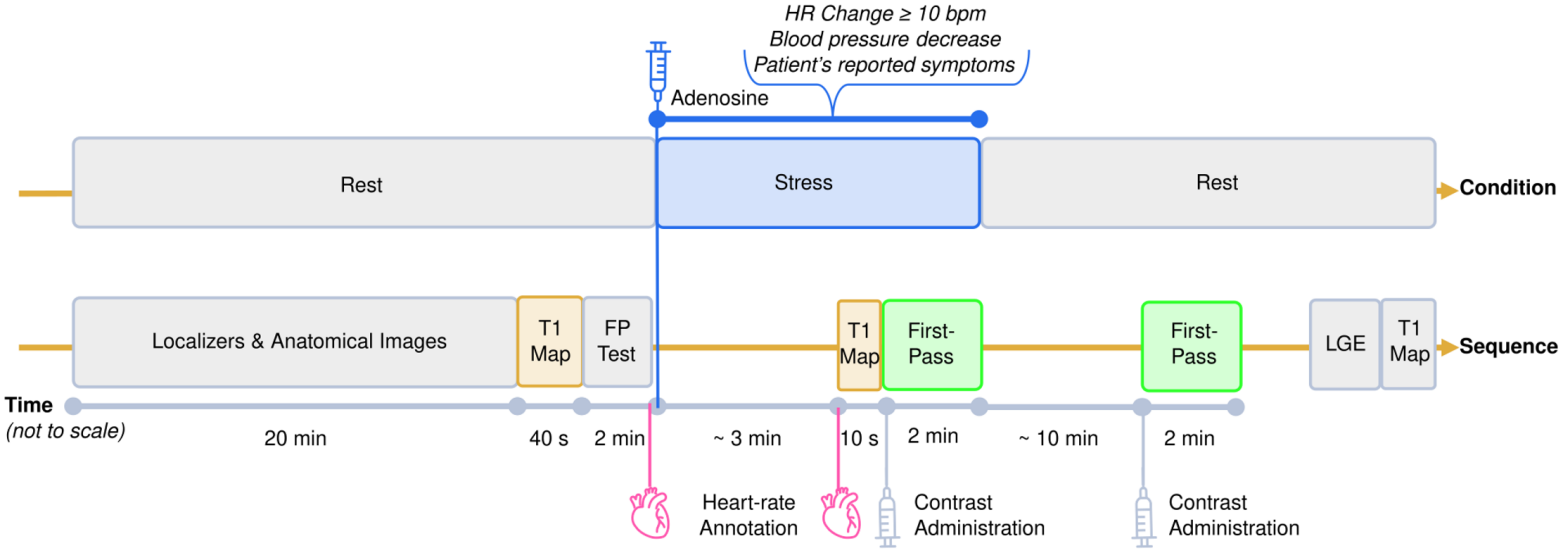
CMR protocol timeline. T1 mapping (yellow) was added to the clinical protocol (gray and green) before the acquisition of first‐pass contrast imaging (green). Time for image acquisition is an approximation since it varied due to heart rate differences. Heart rate was recorded (pink).

T1 mapping was performed using a modified Look–Locker inversion recovery (MOLLI) sequence, with a 5(3)3(0) configuration for both rest and stress, acquired as a single breath‐hold [17]. Other imaging parameters were as follows: single‐shot bSSFP readout, FOV = 350 × 298 mm, matrix = 256 × 144, slice thickness = 8 mm, TR/TE = 334/1 ms, and flip angle = 35°. Motion‐corrected T1 maps were automatically generated by the scanner software.

The stress‐rest first‐pass perfusion sequence was an ECG‐triggered bSSFP optimized for perfusion quantification [18]. Images were acquired during 60 heartbeats in three short axis slices (i.e., in order basal, mid, and apical) during free breathing. A gadolinium dose of 0.75 mmol/kg (Gadobutrol, Gadovist 1 mmol/mL, Bayer, Leverkusen) was used for each stress and rest acquisition. Other first‐pass imaging parameters were as follows: 60 images per slice, bSSFP readout, FOV = 370 × 300 mm, matrix = 190 × 120, slice thickness = 8 mm, TR/TE = 143.55/1.03 ms, and flip angle = 50°. First‐pass images during rest were acquired approximately 10 min after stress first‐pass contrast infusion to allow sufficient time for contrast wash‐out.

Finally, LGE images were acquired 10–15 min after rest first‐pass image acquisition. LGE images were acquired with a phase sensitive inversion recovery (PSIR) steady‐state free precession (SSFP) sequence over the entire myocardial short axis stack, or in user‐determined planes to see specific areas of the myocardium, depending on the criteria of the radiologist present at the study. The inversion time of the LGE sequence was set to null normal myocardium signal to enhance visibility of infarcted areas. Other parameters were: FOV = 360 × 300 mm, matrix = 256 × 192, slice thickness = 8 mm, TR/TE = 904/1.19 ms, and flip angle = 50°.

### 2.3 Data Processing

Visual assessment of first‐pass images was carried out by consensus between two expert radiologists, specialized in cardiac MRI (AE and GBA). Patients with a visual attenuation of splenic perfusion between rest and stress images were classified as having experienced splenic switch‐off, whereas if no attenuation was perceived, a failed switch‐off was registered. This visual assessment was considered as the reference standard for subsequent analyses. The rationale for this choice was that visual assessment represents the clinical standard by which splenic switch‐off has been defined in prior studies.

Additionally, a semi‐quantitative analysis of splenic perfusion was performed based on the signal intensity curves extracted from the first‐pass images in both the myocardial and splenic tissue. One region‐of‐interest (ROI) was manually drawn over a single motion‐corrected high‐contrast intensity image slice to cover the entire visible spleen avoiding partial volume effects from large splenic blood vessels and borders of neighboring tissues. Rest and stress time‐intensity curves were generated from these images. These curves were filtered with a moving average filter (smoothing factor = 0.05), and the maximum value after contrast administration was selected. The maximum value obtained was normalized by subtracting the mean baseline signal. The mean baseline signal was calculated by averaging the intensity values of all the images acquired before the arrival of the contrast to the tissue. The normalized peak value was used as semi‐quantitative measurement of splenic perfusion. Finally, the normalized percentage difference in the signal intensity of the spleen (ΔSI_spleen_) was calculated using Equation 1.

(1) $$∆{\mathrm{SI}}_{\text{spleen}}\left(\%\right)=\frac{\left(\text{Stress}\;{\mathrm{SI}}_{\text{spleen}}-\text{Rest}\;{\mathrm{SI}}_{\text{spleen}}\right)}{\text{Rest}\;{\mathrm{SI}}_{\text{Spleen}}}\times 100$$

The difference in heart rate (ΔHR) from stress to rest (i.e., HR_stress_–HR_rest_) was computed.

In the T1 maps, splenic T1 values from rest and stress were obtained from manually drawn ROIs by two independent researchers (SSB and MFS). For each measurement, the ROIs were drawn covering the visible splenic tissue avoiding partial volume effects from large splenic blood vessels and borders of neighboring tissues. In some cases, artifacts due to failed motion correction were also omitted from the ROIs, especially in the splenic tissue borders. The researchers were blinded to subject clinical data and the T1 map condition (i.e., rest or stress). The T1 values from one of the readers are reported and analyzed in the results. The other measurements were only used to evaluate the interobserver agreement of the splenic T1 measurements. The difference in the T1‐value from stress to rest (ΔT1) (i.e., stress–rest) was calculated.

Quantitative myocardial perfusion maps were generated automatically by the first‐pass imaging dual‐sequence for the complete myocardium [19]. Automatic segmentation of the myocardium left ventricle short axis and extraction of segmental myocardial blood flow (MBF) were carried out inline by the sequence. Only the data from the mid‐ventricular slice was analyzed. MBF in units of ml/g/min was obtained in rest and stress acquisitions, by averaging the segmental values. Myocardial perfusion reserve (MPR) was calculated as the ratio of the average stress to the average rest MBF (i.e., stress/rest).

All data calculations and image processing were performed using in‐house written code in Matlab (R2020b, Mathworks, Natick, Massachusetts).

Images were evaluated by two experienced radiologists (GB and AE) for the assessment of myocardial ischemia and infarction using commercially available software (CVI42, Circle Cardiovascular Imaging, Calgary). Patients were classified as “non‐CAD control” if no signs compatible with ischemia or infarction were found on imaging, or “CAD” if signs compatible with ischemia or infarction were described. This classification was based on the findings of routine radiological evaluation using first‐pass rest and stress images, cine dynamic images, T1 and T2 maps at rest, and LGE images. Patients with other imaging findings, such as fibrosis in areas outside the mid‐ventricular short axis slice that did not affect perfusion, were considered as “non‐CAD control” for this classification. Patients with other incidental cardiac findings non‐related to CAD were excluded from the analysis. Only patients classified as “non‐CAD control” were used to analyze myocardial perfusion data (MBF and MPR) to avoid including perfusion values potentially affected by clinical conditions. Representative patients' maps and images are shown in Figure 2.

**FIGURE 2:**
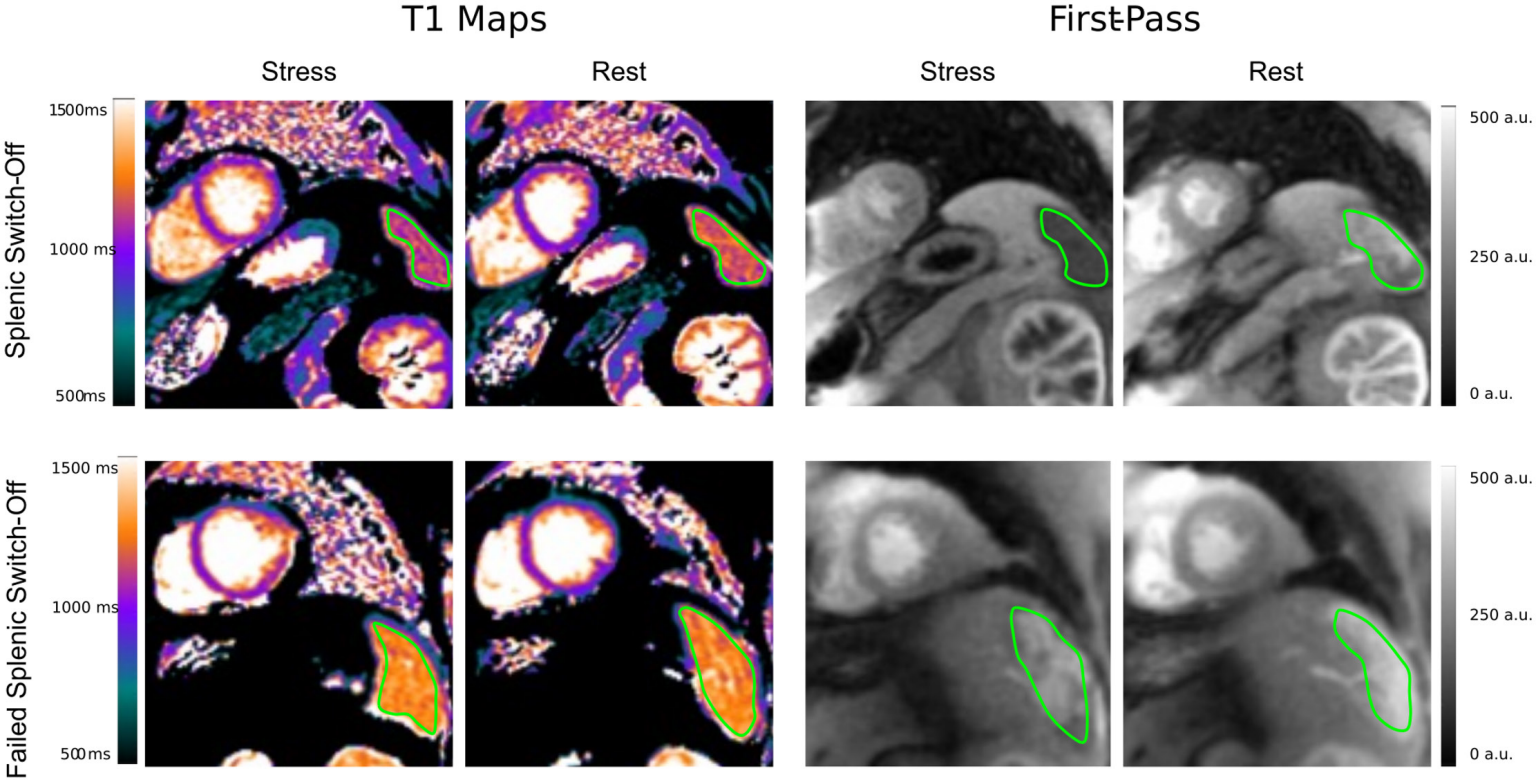
Representative patient with splenic switch‐off and failed switch‐off. T1 map color scale was adjusted to visualize the T1 changes. Green dashed line represents the region‐of‐interest for the spleen measurements. Both patients are non‐CAD controls.

### 2.4 Statistical Analysis

Statistical analyses were performed using RStudio (RStudio Team, Boston, Massachusetts). A *p* value < 0.05 was considered statistically significant. Shapiro–Wilk test was used to evaluate data normality.

First, splenic T1 measurements (rest T1, stress T1, and ΔT1) from the two observers were evaluated for the assessment of interobserver agreement using Bland–Altman plots. The normal distribution of the differences was verified with a Shapiro–Wilk test. The limits of agreement were calculated as the mean difference plus and minus two times the standard deviation of the differences.

Subsequently, splenic T1 values were analyzed to assess differences between rest and stress conditions in switch‐off and failed switch‐off groups. As these data did not follow a normal distribution, an alignment and raking procedure was employed using the aligned rank transform (ART) [20] followed by a mixed factorial analysis of variance (ANOVA) on the aligned data, with two factors: a within‐subject factor named condition with two levels (rest and stress) and a between‐subjects factor named group with two levels (switch‐off and failed switch‐off), and their interaction. When a significant interaction was found in the ANOVA between the two analyzed factors, post hoc contrasts were performed to compare rest and stress conditions within each group, and to compare the groups within each condition (i.e., switch‐off vs. failed switch‐off during rest and switch‐off vs. failed switch‐off during stress) using the Aligned Rank Transformed Contrast (ART‐C) [21] test with Bonferroni correction for multiple comparisons. Confidence intervals for median differences in the original measurement scale were estimated using the exact Wilcoxon–Mann–Whitney test with the Hodges–Lehmann estimator. The exact distribution was selected given the small sample size of the Failed Switch‐Off group (*n* = 7 for MBF and heart rate analyses; *n* = 12 for splenic T1 analyses).

In addition, ΔT1 and ΔSI values were compared between failed switch‐off and switch‐off groups using Wilcoxon rank‐sum tests, and the correlation between ΔSI and ΔT1 values was studied using Spearman's rank correlation. The obtained coefficient of correlation Spearman's rho (ρ_s_) was interpreted as very weak (0–0.19), weak (0.2–0.39), moderate (0.4–0.59), strong (0.6–0.79), and very strong (0.8–1).

Receiver operating characteristic (ROC) curves were generated for ΔT1 and ΔSI to evaluate whether these parameters could detect splenic switch‐off, using the results of the visual assessment of first‐pass images as reference. For each ROC curve, the area under the curve (AUC) was calculated and the optimal threshold for detecting the switch‐off was computed using the Youden index. Sensitivity and specificity values at the optimal threshold were computed. The differences between the ROC curves were studied using DeLong's test for two correlated ROC curves.

Furthermore, myocardial perfusion data (MBF and MPR) measured in non‐CAD controls only were analyzed to evaluate hyperemia. Differences in MBF between rest and stress conditions in switch‐off and failed switch‐off groups were assessed using a 2‐factor ANOVA as described above. MPR was also compared between failed switch‐off and switch‐off groups using a Wilcoxon rank‐sum test.

Finally, to assess the validity of splenic switch‐off as an indicator of myocardial hyperemia (defined as MPR ≥ 1.5) a confusion matrix was computed. Patients with MPR ≥ 1.5 and visual splenic switch‐off (used in this study as the reference standard) were considered true positives and patients with MPR < 1.5 and failed splenic switch‐off true negatives. Sensitivity, specificity, and accuracy were computed. A second confusion matrix was calculated with the heart rate change from rest to stress (ΔHR) as predictor of hyperemia, since ΔHR > 10 bpm is often clinically used as an indicator of adequate myocardial stress response.

## 3 Results

A total of 59 patients with suspected CAD were scanned with the proposed protocol. Two patients could not complete the scan. Four other patients were excluded due to conditions such as myocarditis, acute myocardial damage, or amyloidosis, as these conditions fell outside the scope of this study. A total of 53 patients (40 males) were studied. Patients mean age was 66 years (standard deviation = 13 years).

The spleen was not visible in six patients, who were therefore excluded from splenic analysis. In the remaining 47 patients, no incidental splenic findings were identified. From these 47 patients, 74.5% (*n* = 35) experienced splenic switch‐off according to the expert visual assessment of the first‐pass images.

Among the 47 patients, a total of 28 patients were considered non‐CAD controls. The group of CAD patients (*n* = 19) consisted of five patients with ischemia, six patients with areas of infarction, and eight patients with both conditions present. The final classification of the patients is depicted in Figure 3.

**FIGURE 3:**
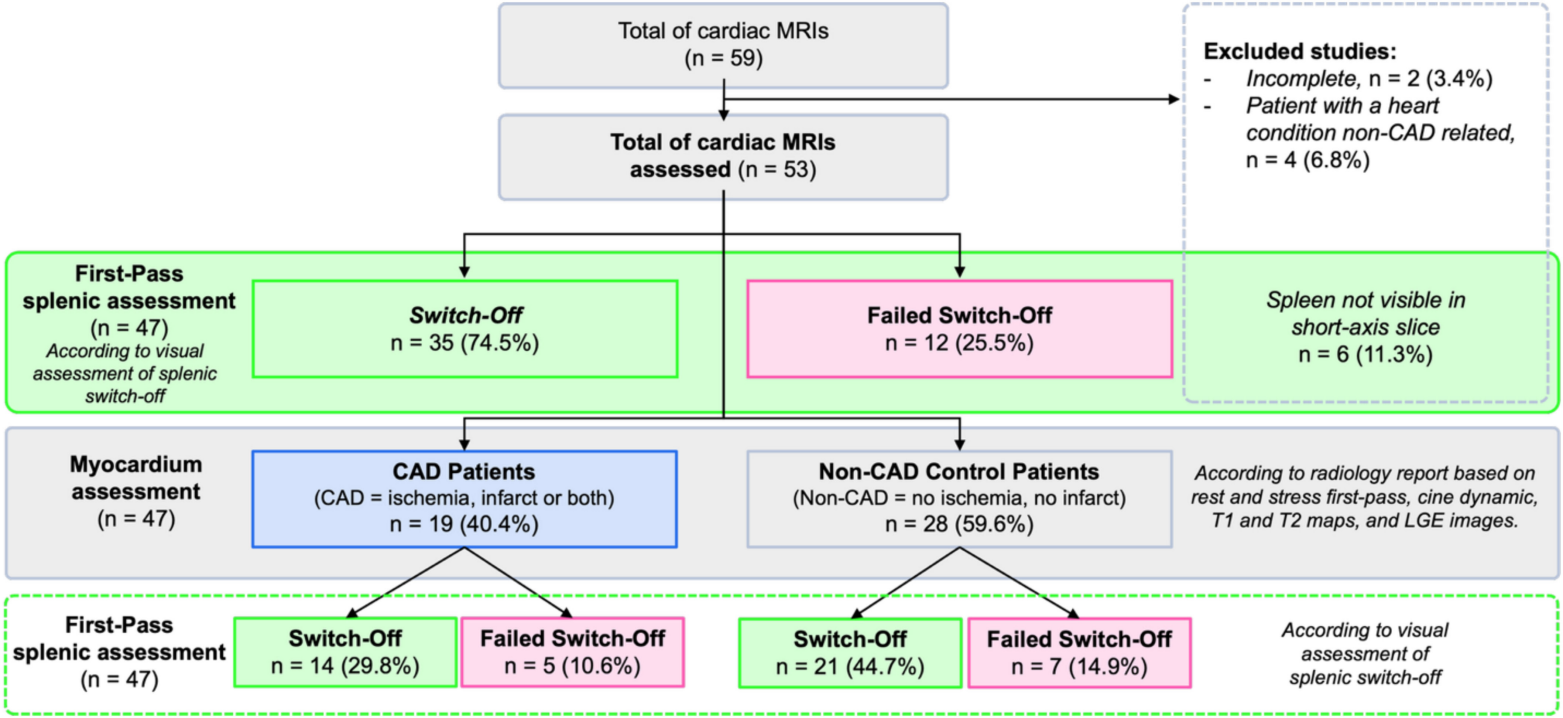
Patient classification according to splenic switch‐off visual assessment and CAD status following stress perfusion CMR. Adult patients with suspected or known CAD who underwent adenosine stress perfusion CMR were classified into two groups based on visual assessment of splenic first‐pass perfusion changes between rest and stress sequences: switch‐off (visible reduction in splenic perfusion at stress) and no switch‐off (absent splenic perfusion reduction at stress). Patients were further categorized as CAD if stress perfusion CMR demonstrated ischemia‐related findings (e.g., perfusion defects, late gadolinium enhancement), or non‐CAD controls if no such findings were identified.

Bland–Altman plots for the interobserver agreement of splenic rest T1, stress T1, and ΔT1 are presented in Figure 4. The limits of agreement for T1 at rest were from −8.91 to 16.43 and from −8.04 to 13.69 at stress. The limits of agreement for ΔT1 were from −15.74 to 13.45.

**FIGURE 4:**
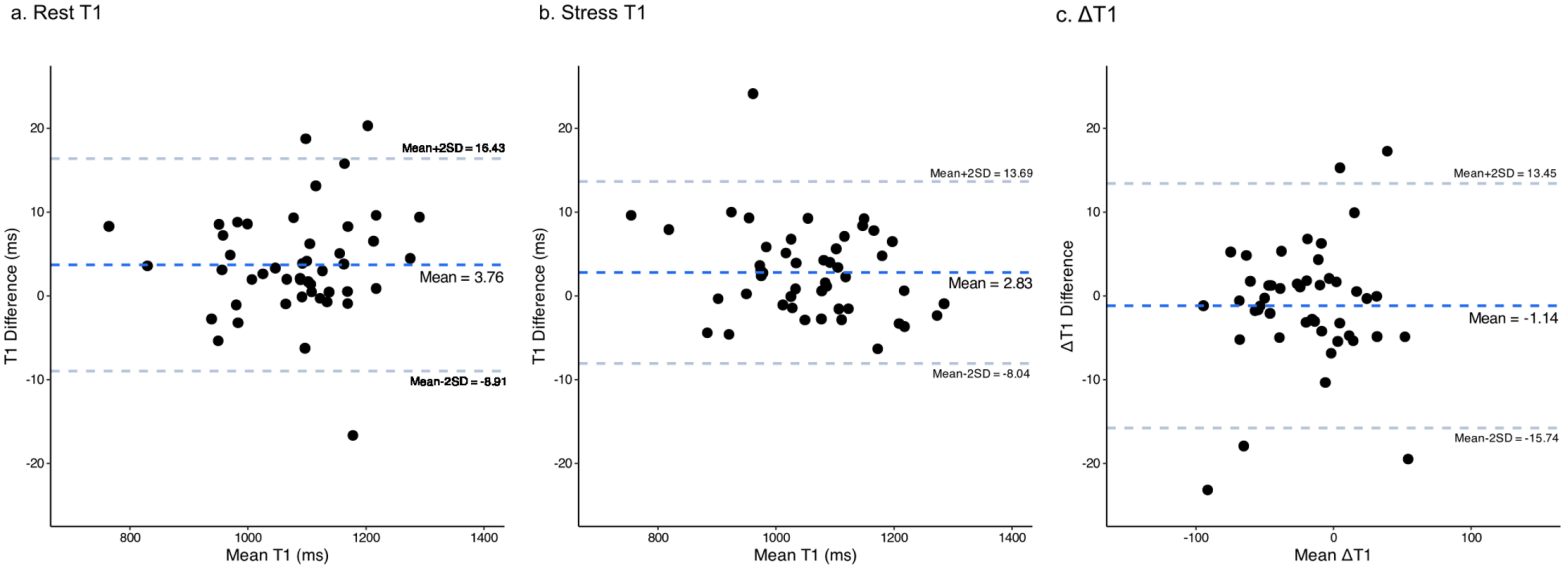
Bland–Altman plots for the interobserver agreement of the splenic T1 measurements made by two independent researchers.

Values of the measured variables (Rest T1_spleen_, Stress T1_spleen_, ΔT1_spleen_, and heart rate) are reported in Table 1, for each group (switch‐off and failed switch‐off) and condition (rest and stress).

**TABLE 1:** Results from patients in switch‐off and failed switch‐off groups.

	Group			
	Switch‐off	Failed switch‐off	*p*	CI (95%)
*n*	35	12		
Age (years)	65 (57, 75.5)	71 (66.5, 75.5)	0.28	(−3, 13)
Sex (male/female)	28/7	7/5		
Rest T1_spleen_ (ms)	1094.1 (992.3, 1147.8)	1105.4 (1078.1, 1165.7)	1	(−40.1, 100.7)
Stress T1_spleen_ (ms)	1029.0 (974.9, 1119.3)	1099.2 (1077.3, 1167.3)	0.26	(−0.76, 140)
ΔT1_spleen_ (ms)	−35.2 (−56.9, −9.2)	10.3 (−7.7, 33.1)	**0.001**	(18.2, 65.7)
ΔSI_spleen_ (%)	−42.2 (−60.9, −34.8)	21.5 (−7.9, 37.8)	**<0.001**	(40.4, 102.7)
*Non‐CAD controls*				
*n*	21	7		
Rest MBF (mL/g/min)^a^	0.8 (0.7, 0.9)	0.7 (0.6, 1.0)	1	(−0.2, 0.2)
Stress MBF (mL/g/min)^a^	2.0 (1.9, 2.9)	1.1 (0.9, 2.1)	**0.04**	(−1.3, −0.1)
MPR*	2.7 (2.1, 3.3)	1.9 (1.2, 2.6)	0.10	(−1.7, 0.1)
Rest HR (bpm)^a^	66 (60, 71)	58 (49, 63)	0.55	(−20, 1)
Stress HR (bpm)^a^	85 (76, 100)	78 (60, 85)	0.05	(−34, 0)
ΔHR^a^	21 (15, 30)	15 (4, 22)	0.07	(−20.1, 0.9)

*Note:* Data are presented as median and interquartile range Q_2_ (Q_1_, Q_3_). The 95% confidence interval (CI) of the differences between failed switch‐off minus switch‐off groups in the different conditions (rest and stress) was estimated using the Hodges–Lehmann estimator via the exact Wilcoxon–Mann–Whitney test. *p* Value: statistical significance of the differences between groups.

Abbreviations: CAD, coronary artery disease; SI, signal intensity.

^a^Myocardial blood flow (MBF), myocardial perfusion reserve (MPR), and heart rate (HR) are only reported for the non‐CAD controls.

In the analysis of splenic T1, there was a statistically significant interaction (*p* = 0.001) between the group (i.e., switch‐off or failed switch‐off) and the condition (i.e., rest and stress). Post hoc contrasts between conditions within each group showed statistically significant differences between rest and stress only for the switch‐off group (*p* < 0.001), where splenic T1 values decreased from rest to stress. Contrasts between groups within conditions were not significant. Data is graphically represented in Figure 5.

**FIGURE 5:**
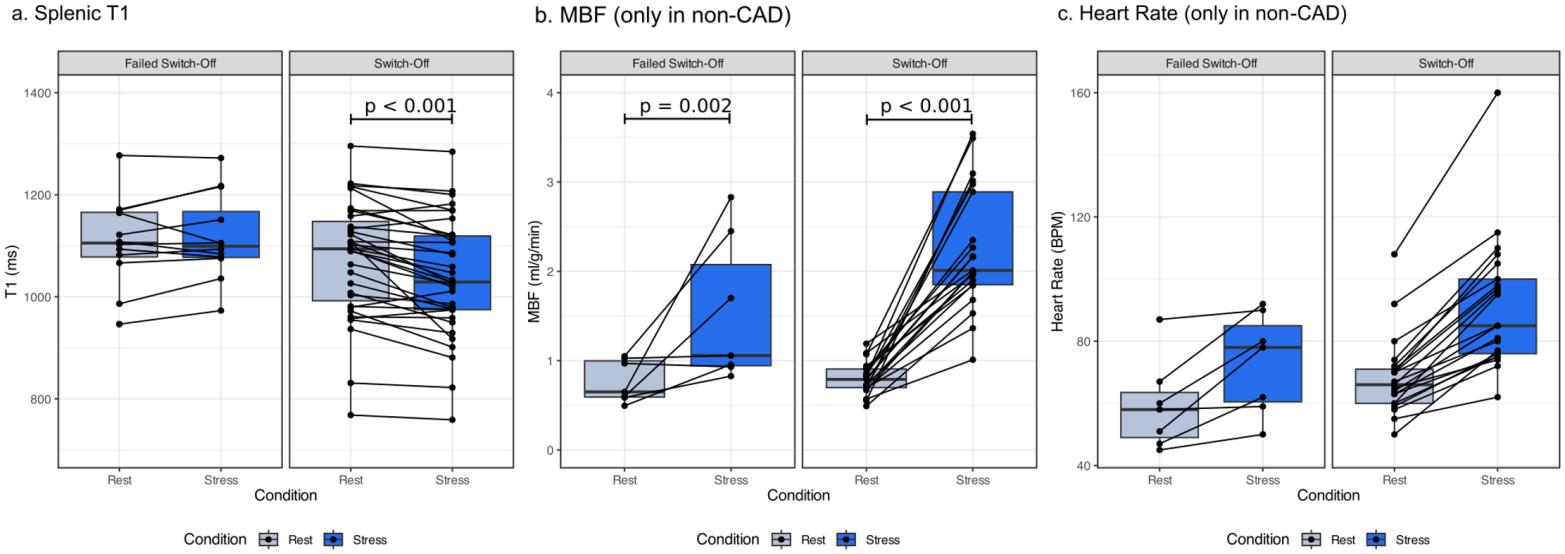
(a) Splenic T1 values. (b) MBF measurements (only in non‐CAD patients). (c) Heart rate (only in non‐CAD patients) for rest and stress in switch‐off and failed switch‐off groups.

The calculated ΔSI_spleen_ showed statistically significant differences (*p* < 0.001) between the failed switch‐off and the switch‐off groups (Figure 6a). For ΔT1_spleen_, a statistically significant difference was also found between groups (*p* = 0.001) (Figure 6b). There was a significant positive weak correlation (ρ_s_ = 0.37, *p* = 0.01) between both techniques (Figure 6c).

**FIGURE 6:**
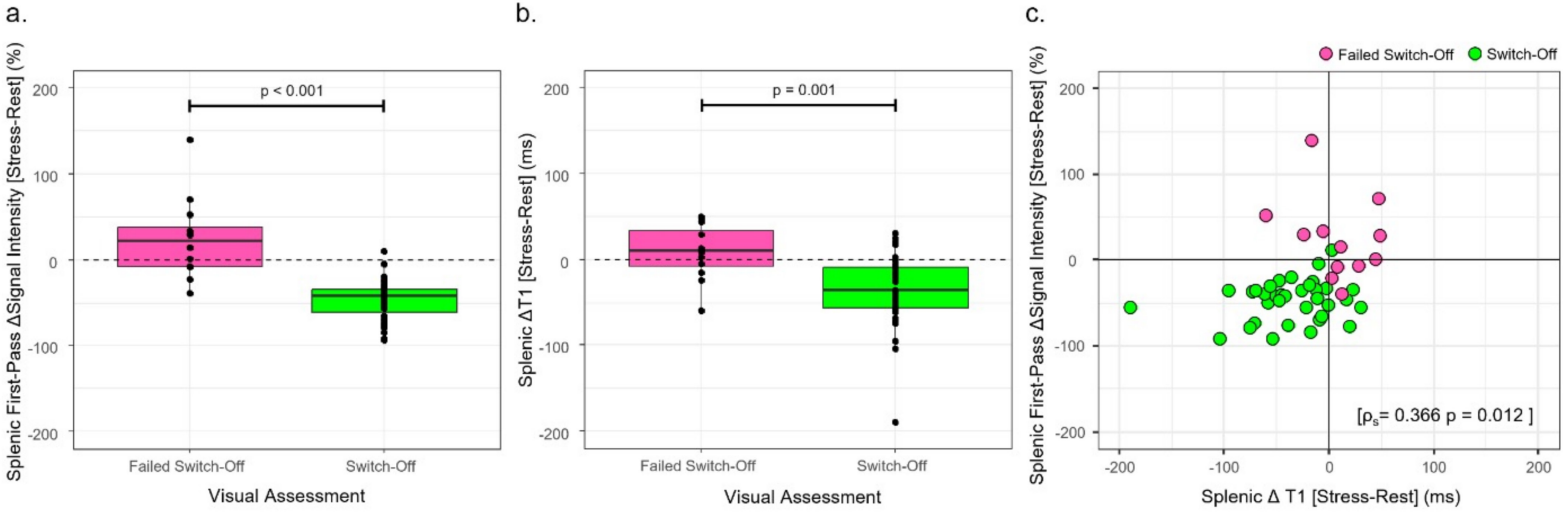
Splenic ΔSI (a) and ΔT1 (b) values for failed switch‐off and switch‐off groups. (c) Scatter plot of ΔSI values and ΔT1 showing the correlation between both techniques.

ROC curves constructed for the two measured variables are depicted in Figure 7. ΔT1_spleen_ classified the failed switch‐off and switch‐off groups with a specificity of 75% and a sensitivity of 80% (AUC 81.2%, 95% CI 66.7%–95.8%). ΔSI_spleen_ classified the group with a 91.7% specificity and a 91.4% sensitivity (AUC 94.9%, 95% CI 88.0%–100%). There were no statistically significant differences between the ROC curves for the ΔT1_spleen_ and ΔSI_spleen_ (*p* = 0.11).

**FIGURE 7:**
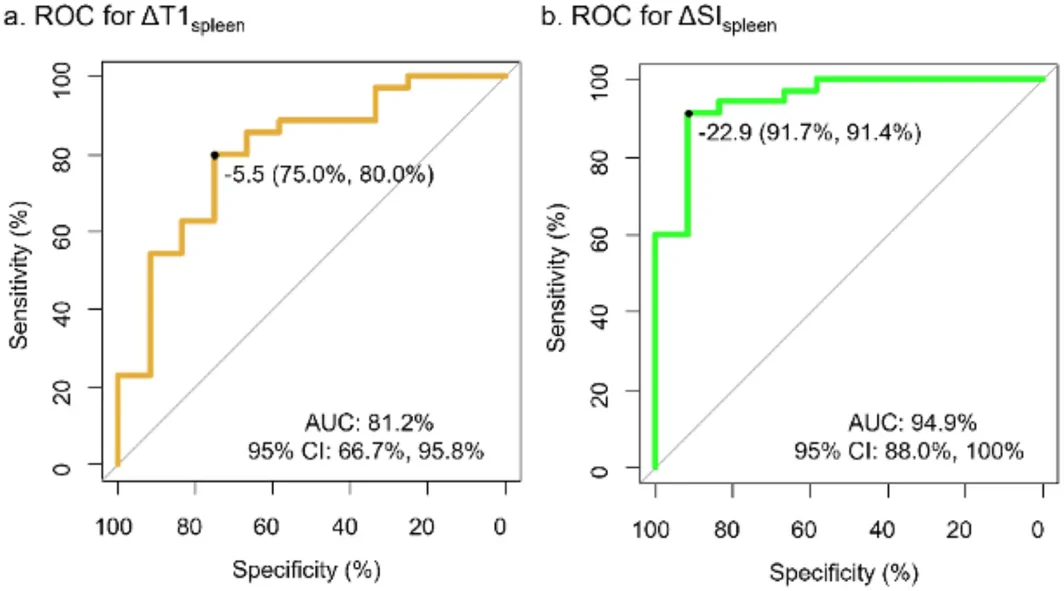
ROC curves for splenic switch‐off classification: (a) ΔT1_spleen_ and (b) ΔSI_spleen_.

In addition, mean MBF values obtained based on the first‐pass quantitative analysis were compared between switch‐off and failed switch‐off groups, including only non‐CAD controls to assess whether the splenic switch‐off reflected the hyperemic state of the myocardium. A statistically significant interaction between condition (i.e., rest and stress) and group (i.e., failed switch‐off and switch‐off) was found (*p* = 0.01). Post hoc tests revealed statistically significant differences in MBF between rest and stress in the switch‐off (*p* < 0.001) and failed switch‐off (*p* = 0.001) groups. In both groups, the MBF increased during stress, but the median stress MBF was higher in the switch‐off group (2.0 vs. 1.1 mL/g/min, *p* = 0.04).

In the MPR comparison between both groups, there were no statistically significant differences between the failed switch‐off and the switch‐off groups (*p* = 0.1). The confusion matrix obtained with the actual condition being MPR > 1.5 and the predictive condition being the switch‐off visual classification resulted in a total of 21 true positives, 3 true negatives, no false positives, and 4 false negatives. This evaluation showed an accuracy of 0.86, a sensitivity of 0.84, and a specificity of 1. The confusion matrix obtained with the heart rate change (ΔHR > 10 bpm) as predictive condition resulted in a total of 22 true positives, 2 true negatives, 1 false positive, and 3 false negatives. This evaluation showed an accuracy of 0.86, a sensitivity of 0.88, and a specificity of 0.67. Data is graphically represented in Figure 8.

**FIGURE 8:**
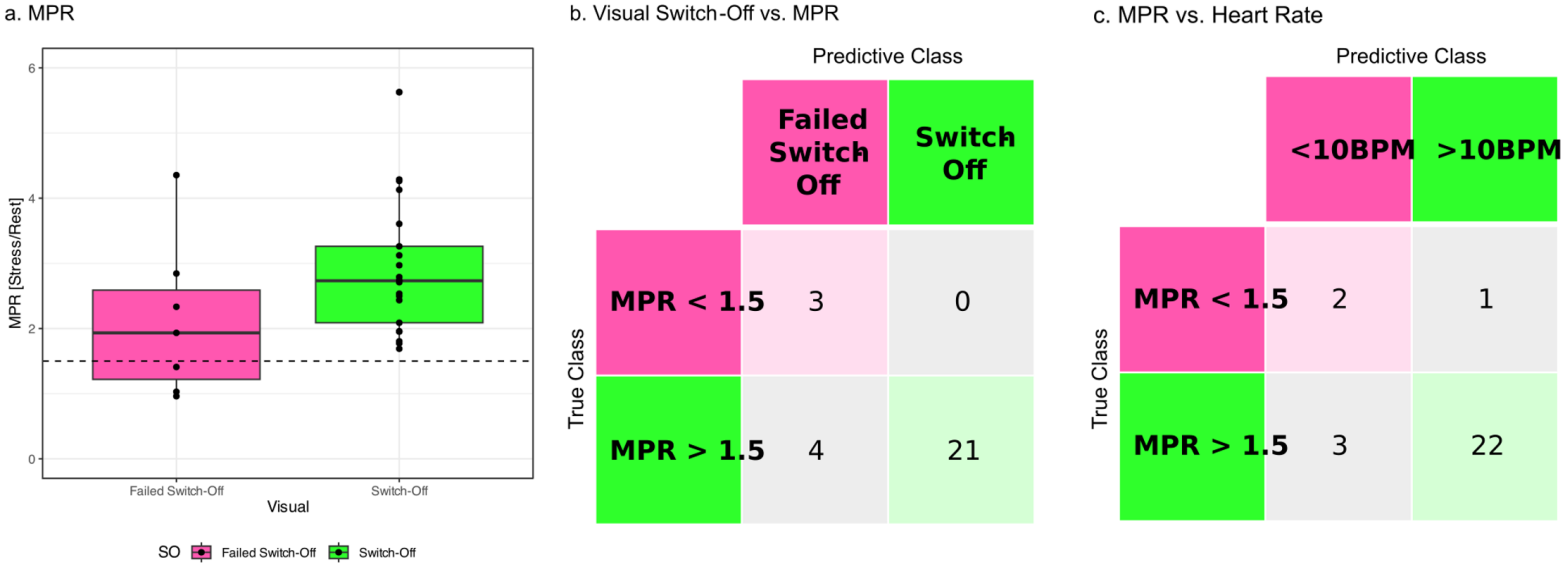
MPR results in non‐CAD controls and confusion matrix.

## 4 Discussion

Splenic T1 values decreased from rest to stress in patients who experienced splenic switch‐off, while no statistically significant differences were observed between conditions in patients who did not experience splenic switch‐off. Lower T1 values in the spleen at stress than at rest were likely due to decreased perfusion in the spleen caused by the adenosine‐mediated vasoconstriction [22], since splenic T1 values are sensitive to splenic blood volume and tissue water content and distribution changes [23]. The findings suggest that splenic T1 mapping could serve as an objective, quantitative adjunct to visual SSO assessment for evaluating adenosine response adequacy.

Similar proportions of patients (between 72% and 74%) who experienced splenic switch‐off has been reported in other perfusion studies with a fixed adenosine dose of 140 μg/kg/min [11, 24]. However, in a study with a similar T1 mapping technique, 92% of patients experienced splenic switch‐off apparently with a fixed adenosine dose administered for 4 min [12]. In other studies, with different perfusion techniques and a variable adenosine dose (i.e., increased adenosine dose at 3 min if no heart rate or blood pressure change was experienced), the number of patients who exhibited switch‐off were higher, near 90% [6], 89% [8], and 84.4% [10], showing that a higher dose of adenosine (175 μg/kg/min) may result in a higher proportion of patients who experience splenic switch‐off [25]. However, adenosine doses higher than 140 μg/kg/min have been reported not to induce greater coronary vasodilation [26]. Additionally, higher doses of adenosine do not overcome other situations that may interfere with the effect of adenosine, such as caffeine intake [27] or some medications (e.g., aminophylline or theophylline).

In this investigation, median splenic native T1 values were similar to previously reported T1 values at 1.5 T [28]. Splenic T1 measurements were in good agreement between observers, with narrower limits of agreement in the ΔT1_spleen_ than those previously reported (ΔT1_spleen_ limits of agreement between −31 and 42) [16]. When measuring the change from rest to stress in the splenic T1 (i.e., as ΔT1_spleen_) and in first‐pass signal intensity (i.e., ΔSI_spleen_), statistically significant differences were found between switch‐off and failed switch‐off groups, as previously reported [6, 12, 16].

In the paper by Liu et al. [12], the authors reported that a decrease ≥ 30 ms from rest to stress in the splenic T1 was the optimal threshold to identify patients with splenic switch‐off with a sensitivity of 90% and a specificity of 88%. However, the authors of that study later published an editorial expression of concern stating that the reported splenic signal intensity change (i.e., ΔSI_spleen_) and the spleen T1 change (i.e., ΔT1_spleen_) could not be independently reproduced, neither were they able to reproduce their original results that showed that ΔT1_spleen_ could identify the splenic switch‐off sign [15]. Nonetheless, in a separate study conducted at another center, the authors obtained a similar value of ≥ 28 ms reduction in splenic T1 with similar sensitivity (93%), but lower specificity (68%), suggesting center and protocol‐dependent variability [13]. In this study, the threshold obtained by the Youden index of ΔT1_spleen_ is notably lower than the threshold reported in the studies mentioned above. However, the obtained median of the ΔT1_spleen_ (−35.2 ms) in the switch‐off group is similar to the values reported in those previous studies [11, 13]. In this study, the obtained threshold demonstrated a lower sensitivity, but a higher specificity. Given the small magnitude of the ≥ 5.5 ms change, further research with a higher sample size is needed to better characterize the magnitude of this cut‐off in detecting splenic switch‐off.

It is important to notice that the study by Liu et al. [12] reported ΔT1spleen values by pooling data acquired at 1.5 T and 3 T, despite acknowledging that resting T1spleen values were significantly higher at 3 T compared to 1.5 T in both controls and patients. Since native T1 relaxation times are known to be field‐strength dependent, with higher absolute values obtained at higher magnetic field strengths, combining measurements from different field strengths may introduce heterogeneity in reported T1 values. These authors partially justified this approach by demonstrating that ΔT1spleen was not significantly affected by field strength. This observation is further supported by subsequent work [16] reporting comparable ΔT1spleen values at 1.5 T, consistent with those reported by Liu et al. In the present study, all acquisitions were performed at 1.5 T, thereby avoiding this potential source of variability in absolute T1 values.

It is also important to note that the MOLLI sequence used in this research differs from the shMOLLI sequence used by Liu et al. [12] Both MOLLI and shMOLLI sequences have been shown to underestimate T1 relaxation times due to a prominent dependency on heart rate (e.g., MOLLI) and a constant underestimation of T1 above 800 ms (e.g., shMOLLI) [29]. Therefore, the cut‐off value obtained in this work should be interpreted in the context of these data.

Given this variability across previously published thresholds, caution is advised when applying specific threshold values across centers. Detailed standardization of MOLLI parameters, ROI methodology, and reference software is essential before ΔT1_spleen_ can be reliably adopted for clinical decision‐making. Additionally, further studies are needed to understand the effects of adenosine in the spleen and if adenosine is causing a reduction in the blood volume in the spleen affecting T1 independently, or if other factors may influence this T1 reduction.

First‐pass ΔSI_spleen_ showed higher sensitivity and specificity than ΔT1_spleen_. However, it can only be assessed once the acquisition is over, and once the patient has received CBGA for the stress evaluation. This is useful to validate the results from a cardiac MRI study but requires the patient to be booked for another cardiac MRI or a different cardiac test if the stress response is not adequate. Thus, splenic T1 may be a useful marker of adenosine stress adequacy that can be assessed in real time during the study, since other hemodynamic responses such as the heart rate cannot reliably assess the stress response alone [5].

The weak positive correlation between ΔT1_spleen_ and ΔSI_spleen_ indicates that while both capture aspects of splenic perfusion change, they may reflect partially different physiological processes. Native T1 changes are primarily sensitive to alterations in tissue composition and blood volume, reflecting the combined effects of splenic vasoconstriction, red blood cell pooling, and changes in extracellular water content. In contrast, first‐pass signal intensity changes are predominantly driven by the kinetics and distribution of gadolinium‐based contrast agents and therefore reflect perfusion and tracer delivery rather than static blood volume alone. Additional factors such as differences in temporal resolution, susceptibility to motion and saturation effects, and the nonlinear relationship between signal intensity and gadolinium concentration may further contribute to the observed discrepancy. Consequently, ΔT1 and ΔSI should be considered complementary rather than interchangeable markers of splenic response to vasodilator stress.

In previous work [10], a splenic switch‐off assessment method was presented using the arterial spin labeling technique to measure splenic perfusion during the CMR study that was able to detect switch‐off with an accuracy of 97%. Even though this technique showed promise to control for false negative results related to the lack of effect from adenosine while doing the study, there are important technical details that need to be overcome before using this technique in the clinical setting.

It is also of importance to notice that, in non‐CAD control subjects, there were no statistically significant differences in the MPR measured in both failed switch‐off and switch‐off groups. It was observed that the mean MBF in the myocardium increased from rest to stress in almost all the patients, although with a statistically significant difference between switch‐off and failed switch‐off groups, and higher stress MBF values in the switch‐off group. The presence of splenic switch‐off is a marker of adequate adenosine hyperemic effect in the myocardium, as indicated by an accuracy of 85.7%, sensitivity of 84%, and specificity of 100%. On the other hand, the heart rate has a similar accuracy and sensitivity, but with a much lower specificity (66.6%). The higher specificity of the splenic switch‐off means that when this sign is present, inadequate hyperemia is highly unlikely, reinforcing its value as a dependable indicator of an optimal adenosine response. In contrast, the heart rate response alone is less reliable, thus integrating the splenic switch‐off assessment as a complementary parameter could enhance the overall assessment of hyperemia.

However, the results also show that the absence of splenic switch‐off does not necessarily indicate insufficient myocardial hyperemia, as suggested by the number of false negatives and lower sensitivity with respect to specificity. This aligns with a recent PET and MRI study [11] concluding that, while the presence of splenic switch‐off is a strong indicator of an adequate adenosine response, its absence alone should not be interpreted as failed vasodilatation.

Importantly, given that current clinical protocols rely almost exclusively on heart rate to judge hyperemia, the addition of splenic switch‐off offers an objective and potentially more robust complementary marker, strengthening the overall assessment.

This study is limited by the relatively small sample size, which may have reduced statistical power, especially in the subgroup analysis in non‐CAD controls.

## 5 Conclusion

This work shows the potential of splenic T1 values to identify splenic switch‐off during adenosine stress CMR in a clinical population with known or suspected coronary artery disease.

## Author Contributions

**Sergio M. Solis‐Barquero:** conceptualization, data curation, formal analysis, investigation, methodology, software, validation, visualization, writing – original draft preparation. **Ana Ezponda:** conceptualization, data curation, formal analysis, investigation, validation, writing – original draft preparation. **Verónica Aramendía‐Vidaurreta:** formal analysis, investigation, validation, writing – review and editing. **Marta Vidorreta:** conceptualization, formal analysis, validation, writing – review and editing. **Marina Pascual:** conceptualization, methodology, writing – review and editing. **Gorka Bastarrika:** conceptualization, methodology, resources, supervision, writing – review and editing. **Maria A. Fernández‐Seara:** conceptualization, methodology, resources, project administration, supervision, formal analysis, writing – review and editing.

## Funding

This study has received funding by Spanish Ministry of Science and Innovation (grant: PI21/00578), Sergio Solis‐Barquero received PhD support from Fundación Carolina and Universidad de Costa Rica, and Verónica Aramendía‐Vidaurreta received grant support from Siemens Healthcare, Spain.

## Ethics Statement

Written informed consent was obtained from all patients in this study. Institutional Review Board approval was obtained.

## Conflicts of Interest

Marta Vidorreta is an employee of Siemens Healthcare in Spain. The other authors declare no relationships with any companies whose products or services may be related to the subject matter of the article.

## Data Availability

The data that support the findings of this study are available from the corresponding author upon reasonable request.
